# Sandwich Integration Technique for the Pressure Sensor Detection of Occlusal Force In Vitro

**DOI:** 10.3390/s22010220

**Published:** 2021-12-29

**Authors:** Jinxia Gao, Longjun Liu, Zhiwen Su, Haitao Wang

**Affiliations:** 1 Key Laboratory of Shaanxi Province for Craniofacial Precision Medicine Research, College of Stomatology, Xi’an Jiaotong University, Xi’an 710049, China; gaojinxia@xjtu.edu.cn (J.G.); wanghaitao@stu.xjtu.edu.cn (H.W.); 2 Department of Prothodontics, College of Stomatology, Xi’an Jiaotong University, Xi’an 710049, China; 3 Institute of Artificial Intelligence and Robotics, the School of Electronic and Information Engineering, Xi’an Jiaotong University, Xi’an 710049, China; suzhiwen@stu.xjtu.edu.cn

**Keywords:** sandwich technique, bite force, mechanical stress, embedded circuit, sensors, 3D printing, CAD/CAM

## Abstract

Bite force measurement is an important parameter when checking the function and integrity of the masticatory system, whereas it is currently very difficult to measure bite force during functional movement. Hence, the purpose of this study is to explore the potential technique and device for the measurement and intervention of the continuous bite forces on functional and dynamic occlusal condition. A portable biosensor by sandwich technique was designed, and the validity, reliability, and sensitivity were determined by mechanical pressure loading tests; meanwhile, the pressure signal is acquired by, and transmitted to, voltage changes by the electrical measurements of the sensors. The result is that, when the mechanical stress detection device is thicker than 3.5 mm, it shows relatively ideal mechanical properties; however, when the thickness is less than 3.0 mm, there is a risk of cracking. Mechanical stress changing and voltage variation had a regularity and positive relationship in this study. The mechanical stress-measuring device made by medical and industrial cross has a good application prospect for the measurement of bite force during function.

## 1. Introduction

The masticatory system is generally considered to be made up of temporomandibular joints (TMJ), muscles of mastication, jaws, teeth, and periodontal tissues [1]. Bite force is generated by the coactivation of all the above craniomandibular structures, initially exerted from the application of the muscle force, and then transmitted through the occlusal contacts between the maxillary and mandibular teeth [2]. Any changing of the occlusion may result in the abnormal activities and bite forces [3]. When the load cannot coordinate with the whole masticatory system in maintaining dynamic equilibrium in function, which may damage the jaw system, it ultimately leads to the occurrence of TMJ disorders, orofacial pain, tooth fracture, and other occlusal-related diseases [4]. Currently, the diagnosis and treatment of occlusion is still one of the most difficult problems faced by dental clinics. Maximum bite force (MBF) measurement plays an important role in evaluating the function and efficacy of masticatory system [5,6]. A previous study has reported that the MBF of natural teeth in healthy adult individuals is highest in the molar regions, for which the average level is between 300 and 600 Newtons (N) [7]. Therefore, quantitative detection and intervention of bite force is of great significance for the diagnosis and treatment of occlusal diseases.

Human bite forces have been researched and estimated by various methods according to previously published studies. The bite forces are typically measured by using mechanical devices at the initial stages, meanwhile sensitive electronic, or a combination of both devices with strain-gauges, piezo-electric sensors, and pressure sheets have grown in popularity in recent years [8]. Bite force-recording devices have undergone many changes, and some of them are commercially available, such as the T Scan system (Tekscan Inc., Boston, MA, USA) [9], the Prescale system (GC Co., Ltd., Tokyo, Japan) [10], Flexiforce (Tekscan, South Boston, MA, USA) [8], etc. These particularly designed or modified devices can record intra-oral bite force in real time, which plays an important role in the determination of occlusion examination. Nonetheless limitations of the current bite force devices are still present in that the T Scan system cannot measure the bite force accurately, meanwhile the Prescale system is unable to function continuously. The other various measurement devices are mostly highlighted in experimental studies and warranted further clinical validation [11]. Meanwhile, electromyography has been reported in recently published research as a possible technique for bite force registration [12]. However, the previous research was mainly focused on bite force detection, and there is a lack of in-depth research on how to control these abnormal forces during functional performance in real time. Therefore, research on quantitative bite force detection and intervention devices, making the diagnosis and treatment of occlusal diseases measurable, controllable, and predictable have sparked tremendous interest in recent years.

Recently, the continued progress and combination of medical sensors, the Internet, and artificial intelligence technology attracted attention in intra-oral practice. A dental implant temperature sensor with a microfabrication process has been proposed for long-term monitoring in the oral environment [13]. It shows that the microscale implantable temperature sensor can stably conduct real-time measurements of temperature changes at the site of dental implants to send warning signals when inflammation occurs. For in vivo oral health monitoring, various sensors have been integrated into denture, orthodontic braces, and mouth-guard platforms to detect humidity for orthodontic bond failure diagnosis and salivary metabolites [14,15]. A poly(*N*-isopropylacrylamide)-based hydrogel was used to develop a tiny tooth-mounted sensor that could calculate temperature change based on its interlayer volume change [16]. Some other studies have summarized various sensor-embedded and wireless teeth sensors for oral activity recognition [17,18,19]. In brief, the emerging sensor technology also brings new opportunities for the quantitative research of bite force. Today, pressure-sensitive, piezoelectric, or piezoresistive transducers are widely used to measure bite force by embedding them in fabricated restorations, such as implants or occlusal splint [6]. Fastier-Wooller et al. presented a low-cost, bite force sensor which can be manufactured in-house by using an acrylic laser cutting machine. It is low cost and simple but cannot be integrated into occlusal splint because it is too thick [20]. Another study from Fernandes et al. also had the same limitation of sample size, especially for the thickness of the detection device [21]. Lantada et al. designed a bite force sensor for monitoring bruxism based on magnetic near-field communication [22]. The inputted bite force signals were then converted into digital signals and transmitted by the wireless Bluetooth to a server terminal [23]. These collected bite force data can be analyzed by deep learning, which is the representative virtual component of AI. We suppose that this kind of smart device can potentially provide quantitative diagnosis and treatment of occlusal disease, meanwhile, it can also carry out remote monitoring, real-time response, and the necessary intervention requirements for a patient’s abnormal occlusal status.

Though bite force measurement is an important parameter when checking the function and integrity of the masticatory system, it is currently very difficult to measure and control the bite force during functional movement. Hence, the purpose of this study is to explore the potential technique and device for the measurement and intervention of the continuous bite forces on functional and dynamic occlusal condition. In addition, the sensitivity and reliability of the designed and manufactured device are evaluated during the mechanical force function.

## 2. Materials and Methods

In this study, we proposed a portable biosensor for the quantitative detection of mechanical force in vitro by using a computer-aided design and computer-aided manufacture (CAD/CAM) technique and 3D printers. The validity, reliability, and sensitivity were determined by mechanical pressure loading tests, meanwhile, the pressure signal was acquired and transmitted to voltage changes by the electrical measurements of the sensors. This study was composed of different experiments, as follows:

### 2.1. Portable Biosensor Design by Sandwich Integration Technique

A portable biosensor for quantitative detection of bite force was specifically designed and fabricated by using the sandwich integration technique (shorted to sandwich technique). The main body of this device consisted of three layers: upper, intermediate, and lower, of which the upper and lower layers fabricated with dental resin materials, whereas the intermediate layer was built up with both resin and a piezoresistive-film sensor. The sandwich technique was outlined in a previously published article [23]. Meanwhile, future exploration and improvements have been conducted based on the foundation of our preliminary research. The ultimate goal of this series’ research is to design and manufacture a bite force measurement device that can be worn in the mouth. The force detection site is located on the occlusal surface of the U-shaped dentition (such as the first molars on both sides), and the battery and the control chip are located on the palatal section of the upper jaw.

#### 2.1.1. CAD Designing and 3D Printing

**Figure 1:**
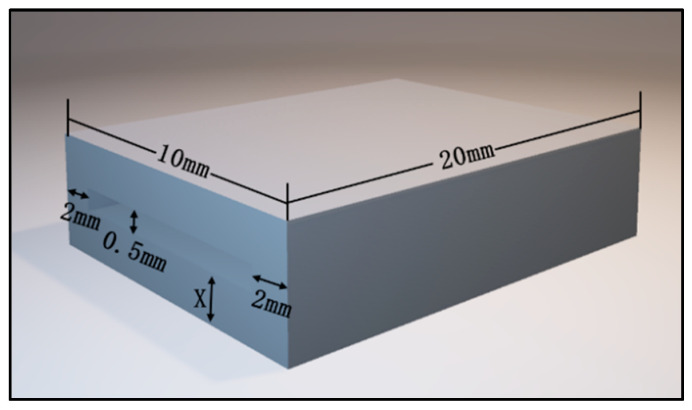
Three-dimensional image exported by CAD software.

**Figure 2:**
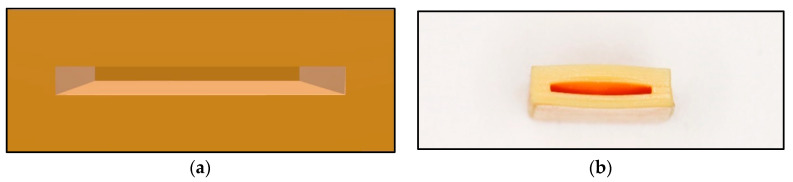
Main body of the detection of bite force device: (**a**) The virtual 3D models; (**b**) Printed objects.

The fabrication details of the device for bite force detection on the local position are as follows: resin samples were designed using computer-aided design (CAD) software (TRIOS 3, 3 Shape, Danish) prior to three-dimensional (3D) printing. The upper and lower layers had the same size of 20 mm in length and 10 mm in width, meanwhile five different thicknesses were set in 0.5 mm, 1.0 mm, 1.5 mm, 2.0 mm, and 2.5 mm, respectively. The experiment result of dimension parameters of the resin samples are listed in Table 1, meanwhile the experiment result of the 3D image by CAD software is shown in Figure 1. The 3D image data were saved in Standard Tessellation Language (STL) file format and then fabricated with resin composite materials (Model V2.0, UnionTech, Shanghai, China) using additive manufacturing techniques and a 3D printer (S300, UnionTech, Shanghai, China). These dental materials are commonly used in dental practice. The experiment result of the virtual 3D models and printed objects are presented in Figure 2.

#### 2.1.2. Conjunction of Resin Specimens and Piezoresistive-Film Sensors

**Figure 3:**
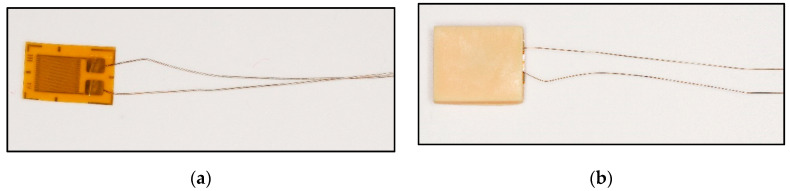
Piezoresistive-film insertion: (**a**) Stress-sensitive chips; (**b**) Stress-sensitive chips can be inserted smoothly.

The main body of the device, created by 3D printing technique, is a hollow cuboid structure with an opening on one side. The prepared film is gently inserted into the main structure of the resin sample. After that step, only the electrical circuit is exposed, and the rest was wrapped inside the resin body. The piezoresistive-film insertion process should be conducted gently and without any resistance interference. The experiment result of piezoresistive-film insertion is shown in Figure 3. If resistance is encountered, which means that the resin material is not fully shaped during the printing process and the 0.5 mm hollow gap is not completely formed, the resin sample should be replaced by a new one in order to avoid damaging the sensor film.

Marginal sealing is another crucial step after film insertion for resin specimens’ and piezoresistive-film sensors’ conjunction. This encapsulation can be implemented in two ways: the first involves using laser welding to weld a 3D-printed resin into a closed state. The ultraviolet light is emitted by a light-curing lamp and irradiates layer by layer of the liquid resin. This operation cures (polymerizes) the liquid resin and results in an excellent edge sealing. The second method is flowable resin sealing by light-curing technique. Flowable resin composites (Z350XT, 3M ESPE, St. Paul, MN, USA) were selected to bond the two ends of the piezoresistive-film sensor (HuaLanHai, BHF350-3AA, Guangzhou, China) with the lower layer and attached the two cured resin layers together in this trial. Then, light-curing was conducted by using an LED lamp (3M, Elipar^TM^ LED Curing Light, Light-curing unit power: 1200 mW/cm^2^, St. Paul, MN, USA) for 40 s. The newly developed flowable resin composite was characterized by low viscosity, high flowability, and resistance to crack propagation [24], hence providing better marginal sealing, which cannot be interfered with the complex environment of the oral cavity when the bite force is applied. Successful conjunction of resin-based dental materials and piezoresistive-film sensor achieves the foundation of occlusal force detection. At this stage, it should be noted that the two wires of the piezoresistive-film sensors should not contact each other, so as not to cause the occurrence of short circuit phenomenon during the later measurement.

### 2.2. Mechanical Pressure Loading Test

Mechanical pressure loading test and electrical measurements of the sensors were conducted in State Key Laboratory for Manufacturing Systems Engineering, Xi’an Jiaotong University, Xi’an, China.

**Figure 4:**
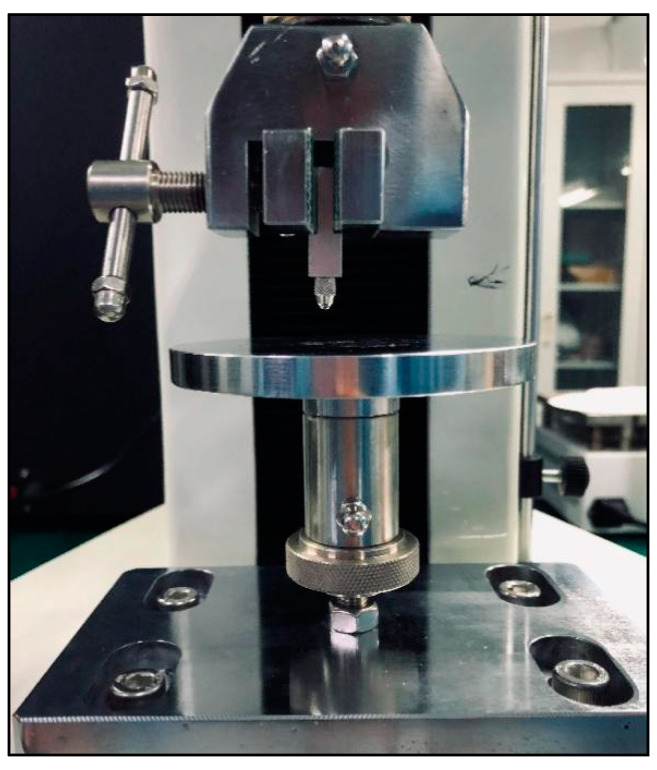
Tensile strength machine and the custom chisel head.

**Figure 5:**
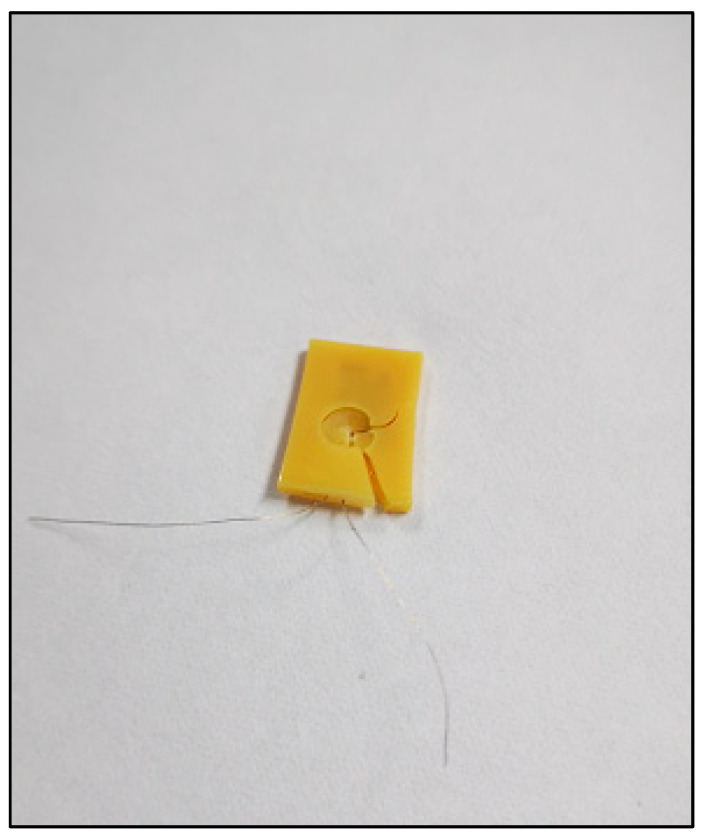
Tensile strength machine and the custom chisel head.

**Figure 6:**
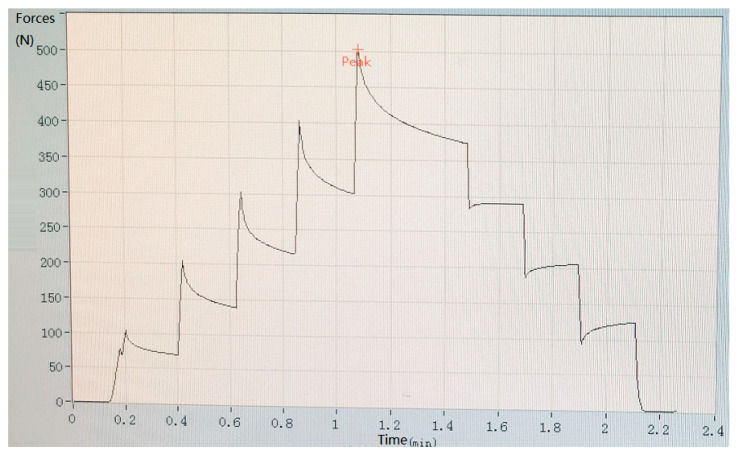
Results of pressure-loading tests when the total thickness of the resin sample is 3.5 mm.

In the experiment, biosensors were divided into five groups based on the different thicknesses of the device’s size: group one (thickness: 0.5 mm); group two (thickness: 1.0 mm); group three (thickness: 1.5 mm); group four (thickness: 2.0 mm); group five (thickness: 2.5 mm). Mechanical pressure-loading tests were conducted according to the computer-aided programs (CI701G) and equipment (tensile strength machine PT-1198, BaoDa, Guangdong, China) for each group sample, respectively; see in Figure 4. The standard mode of force testing is step loading and unloading. Dynamic loading force was set to add 100 N every 12 s by using a custom chisel head (size 2.0 mm), the custom chisel head is presented in Figure 4. The mechanical force involves loading and unloading with a similar procedure, in which the peak value is 500 N. In addition, the stress values loaded by the computer program were also stored in the computer system simultaneously. The experiment results of the pressure-loading tests are illustrated in Figure 5 and Figure 6.

### 2.3. A Customized Pressure Signal Acquisition Controller

**Figure 7:**
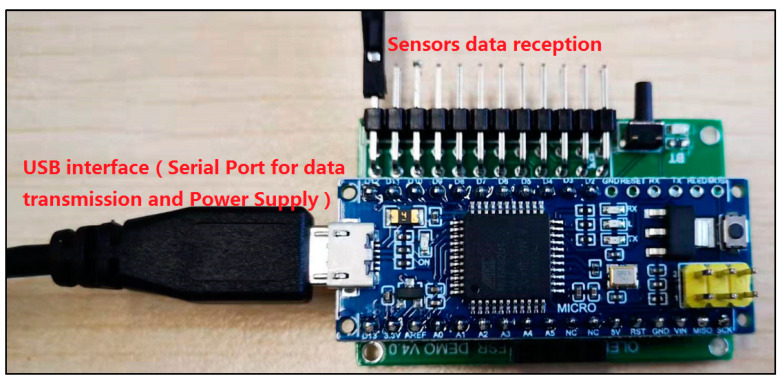
Photograph of the whole system for pressure signal acquisition device.

**Figure 8:**
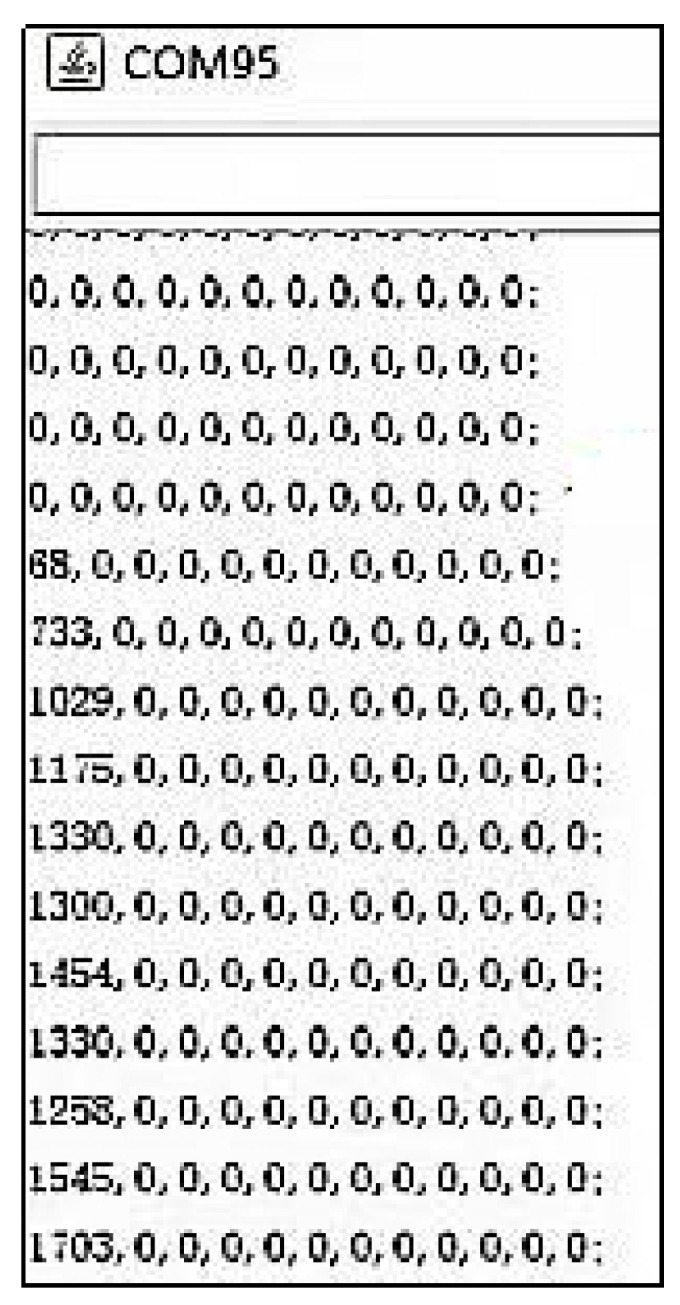
Stress signal acquired from personal computer with protocols of serial port.

**Figure 9:**
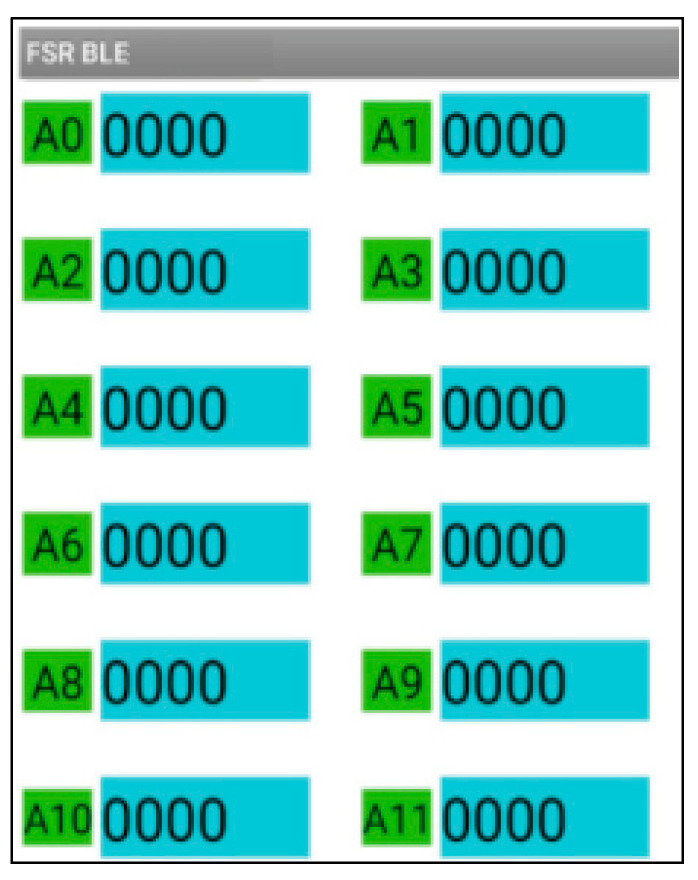
Stress signal acquired from mobile device with protocols of Bluetooth 4.0.

In order to capture the pressure signal of the front-end bio-sensor, a customized pressure signal acquisition controller was leveraged to capture voltage signal. The customized signal acquisition controller can also change the voltage signal to mechanical the stress signal. Moreover, the controller can visualize the stress signal in personal computer or mobile phone by protocols of serial port or Bluetooth 4.0. A picture of the whole controller setup is shown in Figure 7. The controller can support up to 12 channels’ input signal data from film pressure sensors. A low-powered, micro-programmed control unit (MCU) (STC micro, STC89C52, Shenzhen, China) was integrated in the pressure signal acquisition controller. The MCU was programmed and can control the acquisition of 12 channels’ input signal data, converting the analog signals into digital signals through a multi-channel AD (AVIA Semiconductor, HX711 AD, Xiamen, China) conversion module. The converted analog signals were sent to a personal computer or mobile phone for data visualization in real time. The signal represents the range of measuring forces (0~3000 N). If the converted data was sent to personal computer, the protocol of serial port was utilized for data transformation, as shown in Figure 8. This can list the pressure data from the sensors for every 3 s and shows it through a line chart. If the converted data was sent to a mobile phone, the protocol of Bluetooth 4.0 was utilized to accomplish data delivery, as shown in Figure 9. The delivered data can be used for any smart phone APP for data visualization. The power supply of the controller is accomplished by a USB interface (5 V, 1 A). Any standard USB charger can be used for a power supply for the controller.

## 3. Results

### 3.1. Results of Sandwich Technique

A portable biosensor for the quantitative detection of local bite force was designed and manufactured by using computer-aided technology and a 3D printer. The device can be located on the occlusal surface, which is matched with the proximal, distal, buccal, and lingual diameters of human premolars and molars (see in Figure 1 in Section 2.1.1 before). The top and bottom surface were covered by a thin layer of dental resin to change and transmit bite force. The sensitive piezoresistive chip was fixed in the middle layer to collect the mechanical force. The conduction line of the chip was extended outside and was connected to the external equipment to measure the voltage changes (see in Figure 2 and Figure 3 in Section 2.1.1 and Section 2.1.2).

### 3.2. Results of Mechanical Pressure Loading Test

Mechanical pressure loading test was performed by using a tensile strength machine and the custom chisel head (see in Figure 4 in Section 2.2). The maximum values for standard forces, added forces, time interval, and testing time are achieved in this section. After this pressure loading test, we found that the resin sample was prone to fracture when the total thickness was less than 3.0 mm (see in Figure 5 in Section 2.2). Whereas, when the thickness continued to increase, the resin samples showed good flexural resistance. Figure 6 (Section 2.2) shows the mechanical force–timing variation when the total thickness of the resin sample is 3.5 mm.

### 3.3. Results of Electrical Measurements of the Sensors

**Figure 10:**
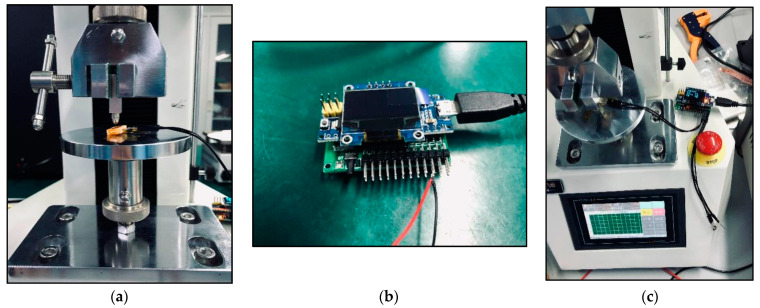
Electrical measurements of the sensors: (**a**) Connection of piezoresistive chip circuit to an external unit; (**b**) Pressure signal acquisition device; (**c**) Voltage changes under mechanical stress loading.

**Figure 11:**
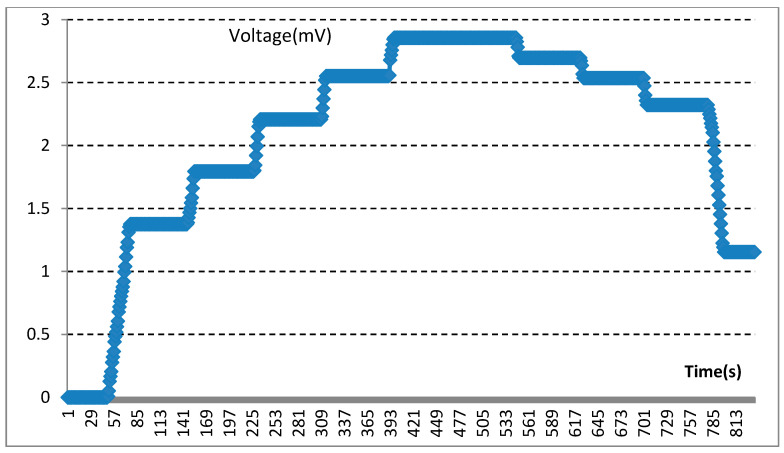
Experimental demonstration for voltage changes.

**Figure 12:**
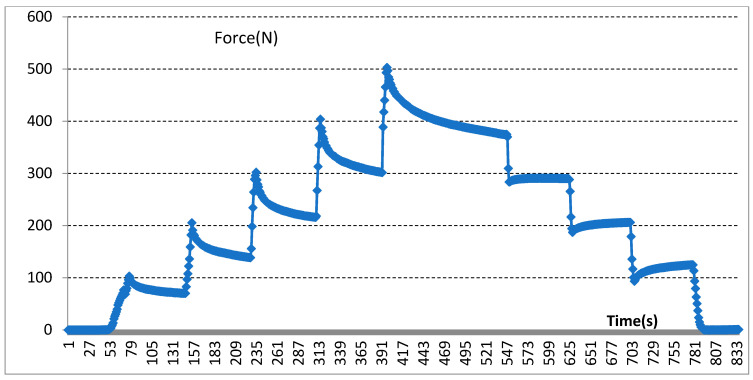
Experimental demonstration for collected occlusal force changes.

Moreover, our various sandwich-integrated sensors were tested in the tensile strength machine, as shown in Figure 10a. The change of voltage from the sensors was acquired by the pressure signal acquisition device, as shown in Figure 10b. Output voltage changes were due to the pressure changes of the tensile strength machine. The voltage signals are transmitted to a personal computer, as shown in Figure 10c. Figure 11 shows the voltage changes with different times. The experiment demonstrates that the trend of voltage changes is the same as the pressure force changes, as shown in Figure 12, which also proves the effectiveness of our sensors and circuits for occlusal force collection.

## 4. Discussion

### 4.1. Main Results

The main finding of this study is that, when the mechanical stress detection device is thicker than 3.5 mm, it shows relatively ideal mechanical properties; however, when the thickness is less than 3.0 mm, there is a risk of cracking. In addition, the electrical measurement unit can detect a positive change in voltage variation when stepping mechanical stress is applied to the sensors. Mechanical stress changing and voltage variation had a regularity and positive relationship in this study, which had great significance in the further measurement of the bite force.

### 4.2. Integration Challenges of 3D Printing Devices and Piezo-Electric Sensors

To date, digital workflows in dentistry involve either subtractive (milling) or additive (printing) processes [25]. With the emergence of digital technologies, 3D printing has become a rapidly developing technology and has been successfully used in the field of dentistry, including prosthodontics, orthodontics, surgical treatment, etc. Three-dimensional printing can be described as an additive manufacturing technique, and, the process of printing can include intra-oral scanning, image acquisition, dental model design with CAD software, computer-aided manufacturing (CAM) by the STL film, and composite resin materials depositing layer upon layer with a 3D printer [26].

The advantage of a 3D approach is that it is more accurate than manual fabrication in object size and thickness, which makes the results of the experiment more credible [27]. Moreover, the low-cost designed resin sheets, ease of manufacturing, time efficiency, and production of intricate details are characteristics of this promising approach, which is useful for experimental testing and clinical application [28]. Although 3D printing technology and piezo-electric sensors have been widely researched and applied in dentistry and engineering fields, respectively, the question of how to effectively combine the 3D printed resin with the engineering chips together is the key and most innovative part of this experiment. Today, 3D printing in dentistry is highly depended on for its the various available material and additive manufacturing (AM) technologies. Despite the standard single-material printers, only a limited number of multi-material 3D printers are commercially available. Multi-material 3D printing with both polymers and metals has been focused on in several studies, and multidisciplinary research is warranted to overcome this limitation [29]. Therefore, embedding the chip into the resin material and forming a good edge sealing in this experiment is full of innovative significance and challenges.

Excellent marginal sealing is the prerequisite for the mechanical stress detection device to function in oral cavity environment. Combination and sealing of the resin sample and sensors are the most difficult procedures in this study. Two kinds of sealing methods were selected in the pre-experimental phase: laser welding and flowable resin sealing by light-curing technique, and the latter was selected in this study. The laser-welding method involves gently brushing a layer of the liquid photo-curable resin polymer in the opening section of the resin sample, and then curing by a UV laser successively, so as to realize the connection and edge sealing of different materials [30]. During the whole pressure test, the sealing edge showed good performance, and there was no separation or stripping between the sealing material and the resin carrier. We will try other more advanced and effective methods to optimize the design in the future.

### 4.3. Detection of Mechanical Signals

The correlation between mechanical forces and voltage changes was analyzed through the data measured in the in vitro experiment. In this experiment, voltage change had presented a corresponding trend with the changing of mechanical stress.

However, the use of different thicknesses of resin material packaging make it difficult for the chip to obtain reliable and sensitive results. The main body of the device should have the ability of high resistance to load under complex conditions. According to previous research, the thickness of the resin layers has an effect on the mechanical force-bearing capacity [31]. The 3D-printed composite resin restorations with a thicker thickness showed higher load to fracture resistance than the thinner ones. It is no surprise that restorations with a higher thickness may disturb or interfere with jaw movement; meanwhile, thinner ones are prone to be easily fractured under high bite forces. For this reason, it is of great significance to evaluate the flexural resistance and sensitivity of the biosensor under different thicknesses. Moreover, the transfer of stress is closely related to the elastic modulus and thickness of the resin sample, which is the mainly topic we need to explore further in the future [32].

### 4.4. Limitations

This paper nevertheless suffers from substantial limitations, such as the fact that the existing detection devices are only able to capture vertical mechanical stress. In fact, simple vertical loading to teeth surfaces has almost never been detected due to complex structures with irregular geometries of natural teeth and the three dimensional movement of the mandible [33]. To some extent, the non-axial bite force which produced by protrusive and bio-lateral movement also plays an important role in the human masticatory system. Therefore, it is vital to improve more optimized sensors and bite force detection systems under varying occlusal conditions. Moreover, the existing measuring equipment will have an influence on the accuracy of force detection due to the thickness of the resin materials. This experiment is at an initial stage in the exploration of an intelligent occlusal detection device. The calculation of force loss, battery assembly, and power consumption involved in the device needs further exploration and research. However, this experiment also provides a new research direction for the exploration of quantitative diagnosis and the treatment of occlusal diseases.

## 5. Conclusions

The present work is a first and very important step in the progression of bite force measurements. In this experiment, the mechanical stress signals show positive correlation with the electrical signals. Calculating the change of the mechanical value through the detection of electrical signals has great significance in measuring the oral bite force. To some extent, the quantitative measurement of bite force by using intricate algorithmic designs and artificial intelligence plays an important role in the diagnosis and treatment of occlusal diseases.

## Figures and Tables

**Table 1:** Reasonable parameters for device for bite force detection on local position.

Item	Parameter		
	Length	Width	Thickness
Upper layer	20 mm	10 mm	0.5 mm, 1.0 mm, 1.5 mm, 2.0 mm, 2.5 mm
Lower layer	20 mm	10 mm	0.5 mm, 1.0 mm, 1.5 mm, 2.0 mm, 2.5 mm
Intermediate (piezoresistive-film sensor)	16 mm	6 mm	0.5 mm (0.3 mm)
Device size	20 mm	10 mm	1.5 mm, 2.5 mm, 3.5 mm, 4.5 mm, 5.5 mm

## Data Availability

Not applicable.
